# Early Functional Rehabilitation after Meniscus Surgery: Are Currently Used Orthopedic Rehabilitation Standards Up to Date?

**DOI:** 10.1155/2020/3989535

**Published:** 2020-03-29

**Authors:** Matthias Koch, Clemens Memmel, Florian Zeman, Christian G. Pfeifer, Johannes Zellner, Peter Angele, Sanjay Weber-Spickschen, Volker Alt, Werner Krutsch

**Affiliations:** ^1^Department of Trauma Surgery, University Medical Centre Regensburg, Regensburg, Germany; ^2^Centre of Clinical Studies, University Medical Centre Regensburg, Regensburg, Germany; ^3^Department of Trauma Surgery, Caritas Hospital St. Josef, Regensburg, Germany; ^4^Sporthopaedicum Regensburg, Hildegard-von-Bingen-Str. 1, 93053 Regensburg, Germany; ^5^Department of Trauma Surgery, University Medical Centre Hannover, Germany; ^6^Sports and Joint Surgery Institute Nuremberg, Nuremberg, Germany

## Abstract

Meniscus therapy is a challenging process. Besides the respective surgical procedure such as partial meniscectomy, meniscus repair, or meniscus replacement, early postoperative rehabilitation is important for meniscus regeneration and return to sport and work as well as long-term outcome. Various recommendations are available. However, the current literature lacks information concerning the actual early rehabilitation in daily routine recommended by orthopedic surgeons. Thus, the purpose of this study was to investigate currently used standard early rehabilitation protocols in the daily routine of orthopedic surgeons. This study investigated the recommendations and concepts for early rehabilitation after meniscus therapy given by German, Austrian, and Swiss orthopedic institutions. Standardized criteria such as weight bearing, range of motion, use of an orthosis, and rehabilitation training were analyzed according to the conducted surgical procedure: partial meniscectomy, meniscus repair, or meniscus replacement. The analysis of standard rehabilitation concepts for partial meniscectomy (*n* = 15), meniscus repair (*n* = 54), and meniscus replacement (*n* = 7) showed significantly earlier functional rehabilitation in all criteria after partial meniscectomy in contrast to meniscus repair techniques (*p* < 0.001). In addition, significant restrictions were found in full weight bearing, full range of motion, and the use of braces. In summary, a wide range of recommendations for weight bearing, ROM, brace therapy, and mobilization is available, particularly after meniscus repair and meniscus replacement. Most concepts are in accordance with those described in the current literature. Further research is necessary to enhance the scientific evidence on currently used early rehabilitation concepts after meniscus therapy.

## 1. Introduction

Meniscus injuries are one of the most common knee injuries overall [1]. Traumatic lesions usually affect athletes and predispose those for the development of an osteoarthritis [2]. Concerning the treatment options, a radical change occurred in the therapeutic strategies of meniscus lesions considering the increasing knowledge about the significant relevance of the menisci for an intact knee joint [3, 4]. As a filling tissue between the femoral condyles and the tibia plateau, the menisci have decisive functional and biomechanical properties and are essential for load bearing, stabilization, proprioception, and lubrication as well as shock absorption in all knee movements [4–8]. In the case of a meniscus lesion, these properties are restricted or even reversed [4, 9]. Thus, loss of meniscus integrity and tissue is associated with increased contact pressure on the articular cartilage, predisposing patients to early joint degeneration [10, 11].

For this reason, there is a consensus to preserve or restore as much meniscus tissue as possible [2, 3, 12]. Due to the low intrinsic regenerative potential of meniscus tissue, however, indications for reparative procedures such as meniscus repair in terms of suturing or meniscus replacement are limited [2, 3]. Thus, the main surgical procedure is still partial meniscectomy [13, 14].

Early postoperative rehabilitation has a crucial impact on postoperative meniscus regeneration and outcome after meniscus therapy [10]. Particularly, meniscus repair as well as meniscus replacement requires special rehabilitation measures for restoring meniscus continuity and an intact knee joint to reduce the risk of developing osteoarthritis in the future and facilitating a safe return to sport and work. However, the current literature does not provide any generally accepted consensus on rehabilitation after meniscus therapy and lacks information about the real daily routine of early rehabilitation recommended by orthopedic surgeons.

Thus, the purpose of this study was to describe the current status of the daily routine of early rehabilitation after meniscus therapy and to analyze if there is a consent concerning the early rehabilitation recommendations of orthopedic surgeons.

## 2. Material and Methods

The written protocols for early rehabilitation after meniscus therapy used in 62 German, Austrian, and Swiss orthopedic institutions were epidemiologically surveyed. These rehabilitation protocols represent standard recommendations by orthopedic surgeons to their patients as well as physiotherapists and include rehabilitation algorithms for the early postoperative period after different types of meniscus surgery. The evaluation of these protocols focused on treatment options, such as partial meniscectomy, meniscus repair, and meniscus replacement. Meniscus replacements included both synthetic and allografts. Conservative treatment options for meniscus lesions were not considered in this survey.

The rehabilitation algorithms were analyzed with regard to postoperative recommendations for weight bearing, restrictions of range of motion (ROM), and brace therapy as well as physiotherapy. Furthermore, criteria for the rehabilitation progress such as the period and time points of limitations were registered. Postoperative weight bearing was divided into 4 categories: no weight bearing (NBW), partial weight bearing (recommendation of any kind of loading up to 20 kg, PBW), half body weight (recommendation of any kind of loading ranging between 20 and 30 kg, HBW), and full body weight (no weight-bearing limitations, FBW). The categories for postoperative ROM were as follows: immobilization 0°, limited flexion of 30° (0-30°), limited flexion of 60° (0-60°), limited flexion of 90° (0-90°), and full range of motion without any limitation. Use of braces was differentiated according to whether such use was recommended or such information was lacking as well as according to the recommended period of use. Recommendations for physiotherapy included the categories of continuous active/passive motion (CAM/CPM) as well as the recommended start of rehabilitation training and/or specific training. In this context, rehabilitation training was defined as basic sport activities, such as ergometer, cycling, aqua jogging, general strength training, or crawling. Specific training included roadwork, coordination, and proprioception training, as well as sport-specific training.

All evaluated categories apart from rehabilitation- or sport-specific training were analyzed up to 3 days postoperatively and 7 days postoperatively followed by weekly intervals until full weight bearing and full range of motion were allowed or an orthosis or further CPM/CAM training was not necessary anymore.

Statistical analysis was done with SPSS® (Version 25, IBM, Armonk, NY, USA). Data are presented as mean (SD) or absolute and relative frequencies. Continuous data were analyzed using the Kruskal-Wallis test with post hoc pairwise comparisons for more than two groups as well as the Mann-Whitney *U* test for two groups. A probability (*p*) value of ≤ 0.05 was considered to be significant for each test. Graphical illustrations were generated with GraphPad Prism® (Version 5.01, GraphPad Software, La Jolla, CA, USA) and Microsoft PowerPoint 2013® (Microsoft Corporation, Redmond, WA, USA).

## 3. Results

**Figure 1:**
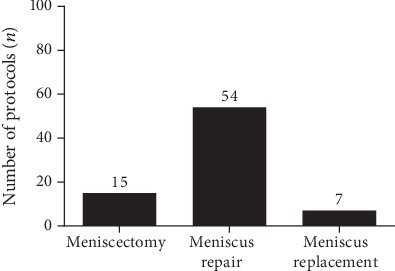
Distribution of rehabilitation protocols after meniscus therapy.

A total of 76 rehabilitation protocols for meniscus therapy were available for this evaluation. The majority (*n* = 54) of protocols referred to postoperative treatment after meniscus repair. 15 rehabilitation concepts were related to partial meniscectomy and 7 recommendations for early rehabilitation to rehabilitation after artificial meniscus replacement (see Figure 1).

### 3.1. Meniscectomy

**Figure 2:**
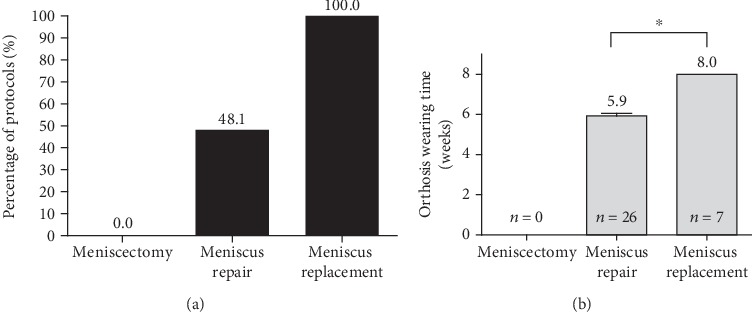
Comparison of meniscus therapy concerning use of orthosis (a) and period of orthosis therapy (b).

**Figure 3:**
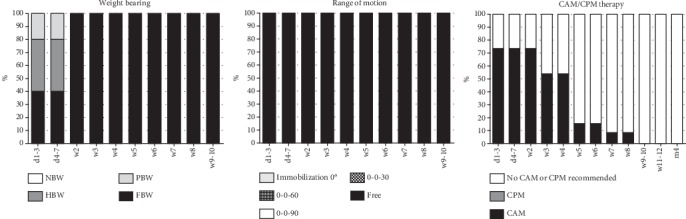
Early rehabilitation after partial meniscectomy including weight bearing, range of motion, and CAM/CPM therapy.

**Figure 4:**
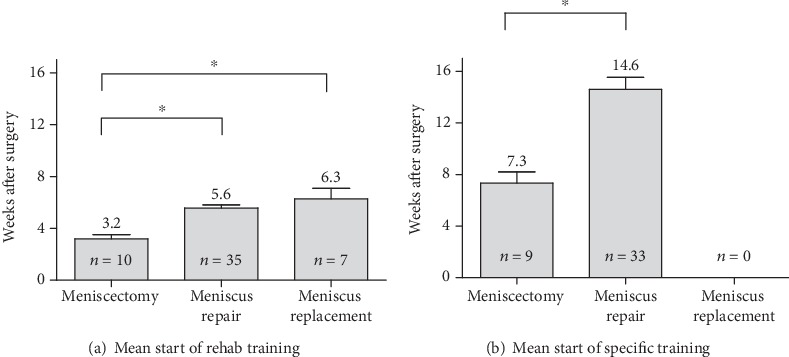
Comparison of recommendations concerning the start of rehab (a) and specific (b) training after surgical meniscus therapy.

The analyzed early rehabilitation protocols show a significantly accelerated rehabilitation after partial meniscectomy. There is a consensus concerning full weight bearing and ROM. Recommendation for full weight bearing start from the second postoperative week (mean: 0.6 weeks; SD: 0.5 weeks; *p* < 0.001 compared to meniscus repair and replacement). ROM is unlimited immediately after surgery in all recommendations. The utilization of orthosis is not supported after partial meniscectomy (see Figure 2). CAM therapy is recommended by the majority of the evaluated protocols for 4 postoperative weeks with an overall mean period of 3.8 weeks (SD: 1.9 weeks; *p* < 0.001 compared to meniscus repair and replacement) (see Figure 3). Supporting rehabilitation training was recommended to start after a mean of 3.2 weeks (SD: 1.0 weeks, *p* < 0.001 compared to meniscus repair and replacement) and specific training after a mean of 7.3 weeks (SD 2.6 weeks, *p* < 0.001 compared to meniscus repair and replacement) (see Figure 4).

### 3.2. Meniscus Repair

**Figure 5:**
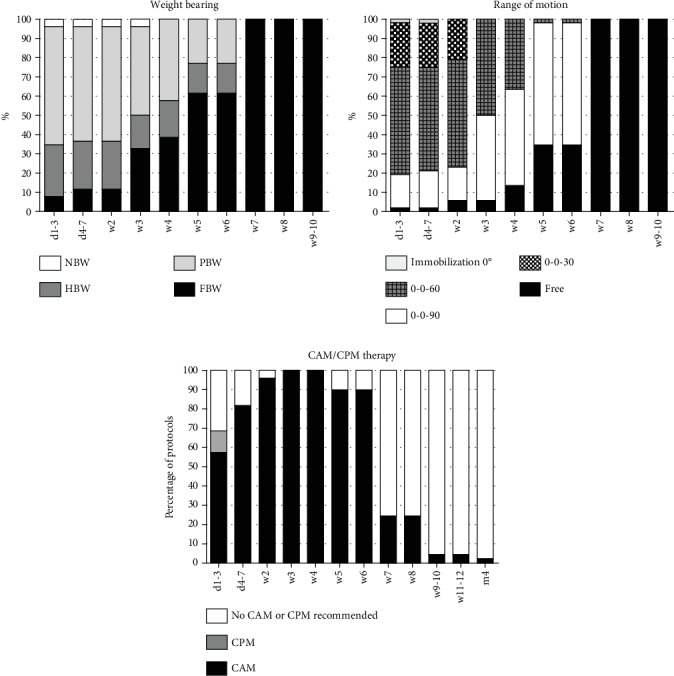
Early rehabilitation after meniscus repair including weight bearing, range of motion, CAM/CPM therapy, and use of orthosis.

The evaluated early rehabilitation protocols show a high variance of recommendations after meniscus repair especially for the first 6 weeks after surgery. Full weight bearing is recommended after a mean period of 3.9 weeks (SD: 2.1 weeks). A complete consensus is available after 6 postoperative weeks. Concerning ROM, there is a consent to allow free ROM after 6 postoperative weeks. Overall, free ROM was significantly earlier recommended in comparison to meniscus replacement procedures (mean: 5.0 weeks; SD 1.6 weeks; *p* = 0.009). External knee stabilization by the use of braces supported almost half of the early rehabilitation concepts (48.1%) for a mean period of 5.9 weeks (SD: 0.7 weeks) (see Figure 2). Restoration of motion using CPM is recommended in 7.4% (*n* = 4) of the assessed concepts limited to the first postoperative week (see Figure 5). In contrast to that, the majority of early rehabilitation protocols recommended the use of CAM for a mean period of 6.4 weeks (SD: 1.9 weeks). Starting rehabilitation training is overall supported after a mean period of 5.6 weeks (SD: 1.5 weeks) and specific training after a mean period of 14.6 weeks (SD: 5.4 weeks) (see Figure 4).

### 3.3. Meniscus Replacement

**Figure 6:**
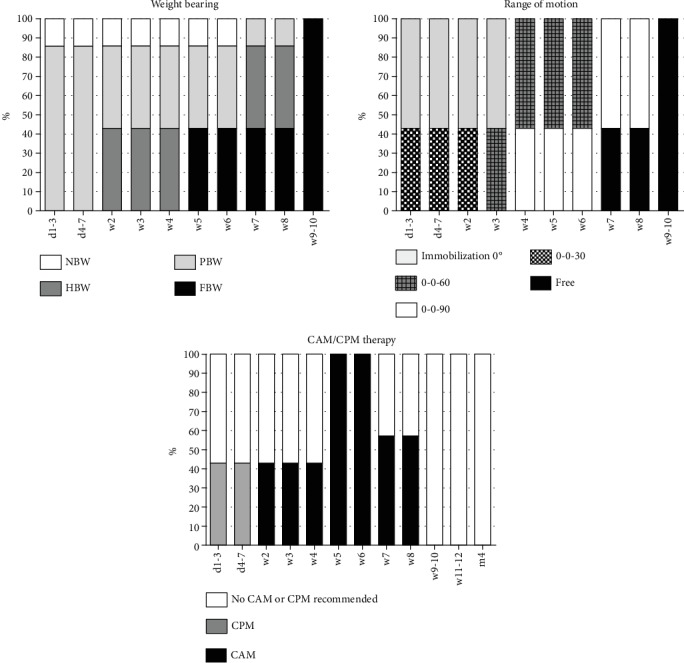
Early rehabilitation after meniscus replacement including weight bearing, range of motion, CAM/CPM therapy, and use of orthosis.

The recommendations for early rehabilitation after meniscus replacement show a high variance and are overall more restrictive in comparison to the recommendations after partial meniscectomy as well as meniscus repair. A complete consensus for full weight bearing exists from the 10^th^ postoperative week. Overall, full loading is recommended after a mean period of 6.3 weeks (SD: 2.1 weeks) and significantly delayed in comparison to meniscus repair procedures (*p* < 0.001). Restoration of ROM is equally significantly delayed in comparison to meniscus repair procedures (*p* = 0.009). Full ROM is recommended after a mean period of 7.1 weeks (SD: 1.1 weeks) and with a complete consent after 9 postoperative weeks. External stabilization of the knee joint using orthosis is supported by all evaluated early rehabilitation protocols for a mean period of 8.0 weeks (SD: 0.00 weeks), what is significantly longer than after meniscus repair (*p* < 0.001) (see Figure 2). The use of CPM is limited to the first postoperative week and 42.9% (*n* = 3) of the evaluated early rehabilitation protocols. In contrast to that, the use of CAM is overall recommended for a mean period of 7.1 weeks (SD: 1.1 weeks) and with a complete consensus for the 5^th^ and 6^th^ postoperative week (see Figure 6). Starting rehabilitation training is overall recommended after a mean period of 6.3 weeks (SD: 2.1 weeks), while specific training is not recommended to start before 6 months after surgery. (see Figure 4).

## 4. Discussion

The present study describes for the first time the variety and differences of currently used early rehabilitation protocols recommended by orthopedic surgeons after different procedures of meniscus surgery. Meniscus therapy is a challenging process, and early rehabilitation is crucial for restoring joint function after surgery. In contrast to partial meniscectomy, which is marked by overall earlier functional rehabilitation and a nearly unrestricted start of weight bearing, full range of motion, and training, the start of early rehabilitation is more restricted after meniscus repair and after replacement of meniscus tissue.

Generally, the main strategies for meniscus treatment are based on the knowledge of tissue engineering and experimental studies. According to the current literature, the endogenous regenerative potential is limited to the peripheral vascularized area of the meniscus but is largely diminished in the central area in correlation with vascularization [5, 15, 16]. Therefore, reparative treatment options are limited to meniscus lesions in the peripheral zone [10]. Also, meniscus replacement with synthetic meniscus implants require an intact meniscus basis and a stable rim of the appropriate meniscus tissue [17, 18]. Thus, partial meniscectomy is still the treatment of choice for many meniscus injuries without an indication for reconstruction, despite the knowledge of the high risk of degenerative long-term changes [10]. Partial meniscectomy leads to fast symptomatic improvement, early functional rehabilitation, and early pain-free return to job or sports. However, early rehabilitation is of significant relevance for each of these treatment options [10, 19, 20].

This study showed a higher number of rehabilitation protocols for meniscus repair than for partial meniscectomy and meniscus replacement. Many orthopedic surgeons conduct both partial meniscectomy and meniscus repair, but meniscus replacement is only offered in very few institutions because of the surgical skills required. According to the quantity and quality of the rehabilitation protocols available, partial meniscectomy seems to be a common procedure, which does not involve any specific restrictions in the early postsurgical period. This investigation also showed that the progress of early rehabilitation after partial meniscectomy was significantly accelerated in all categories. After partial meniscectomy, particularly, effusion and pain are limiting factors for the patients before returning to full activity [10, 21]. Thus, early full weight from the second postoperative week onwards, immediate full ROM, abandonment of any bracing, and use of immediate active motion training are reasonable and in accordance with the current literature [10, 21, 22]. However, muscular and proprioceptive deficits have to be addressed before return to specific training and sports [10]. Many studies have shown muscular deficits particularly of knee extensors which were reduced in comparison to the noninjured limb up to 12 weeks after surgery [10, 23].

In contrast to the early rehabilitation after partial meniscectomy, loading and mobilization progressed carefully after meniscus repair and meniscus replacement. The present study showed a slowly increasing loading after meniscus repair, whereas the percentage of full weight bearing clearly increased from the third postoperative week. Generally, we found a high variety of rehabilitation protocols for meniscal repair, which might be due to the different types of meniscus lesions and the different repair techniques [24]. In addition, other factors such as the individual quality of the meniscus tissue, the localization of the meniscus lesion, and the time of surgery are important factors, which may influence early rehabilitation after meniscus surgery. However, in their literature review regarding the weight-bearing status after meniscus repair, VanderHave et al. described similar good to excellent outcome results for both restricted weight bearing and accelerated immediate weight bearing in the early rehabilitation phase after meniscus repair [25]. They supported the results by Lind et al., who rated free rehabilitation after meniscus repair as safe and not associated with a higher failure rate than after restricted rehabilitation [25, 26]. Furthermore, recent studies have shown a trend towards an accelerated rehabilitation concept with early ROM in addition to early loading of the index limb [25, 27, 28]. In this context, Sherman et al. concluded that protected early ROM is important for meniscus healing and a preventive factor of postsurgical arthrofibrosis [24]. However, the current study yielded a wide variety of recommendations for postoperative ROM, while the majority of protocols recommended restricted knee flexion up to 6 weeks after surgery considering the meaning that immobilization following meniscus repair is essential to meniscus healing [24]. The current literature shows just little evidence for postoperative limitation of knee flexion. Menisci are highly mobile structures between the femoral condyles and the tibia plateau [29], which can impair the healing process after meniscus repair. Lin et al. showed in a cadaveric study that there is no undue stress on meniscal repairs in an unrestricted open chain range of motion [28]. In contrast, the review by Cavanaugh and Killian [30] described a nonbeneficial effect of knee flexion and rotation on the healing process after meniscus repair, what overall rather supports the postoperative restriction of ROM and the use of braces.

The use of an orthosis for a mean period of 5.9 weeks was recommended by almost half (48.1%) of the investigated rehabilitation protocols. However, the current literature only includes few data supporting the utilization of braces. O'Donnell et al. recently reviewed the current literature for rehabilitation protocols after isolated meniscus repair. The authors described a lack of consensus on the use of braces. There is a wide variety of recommendations ranging from 1 to 2 postoperative weeks up to 6 postoperative weeks, for instance in the case of knee extension braces, but without any significant evidence [27]. However, especially in the early rehabilitation period, knee braces protect the healing meniscus tissue by providing a rotational control of the affected leg, respecting the fact that most meniscus injuries occur by combined rotational and flexion forces [24]. Additionally, it compensates initial quadriceps weakness caused by pain or effusion and protect the meniscus repair from deep-flexion weight bearing [24]. Nevertheless, knee motion seems to influence the healing process. In their in vivo studies, Bray et al. described a reduced healing process in injured menisci after immobilization [31]. But, the current literature lacks evidence for the use of CPM or CAM because only one study describes the use of CPM [32]. However, in the currently used rehabilitation concepts, particularly, the use of CAM for early knee mobilization after surgery finds favour with the orthopedics. This study recommended an increase in physical load during rehabilitation training starting 5 weeks after surgery and specific training starting 13 weeks after surgery. In contrast, O'Donnell et al. found in a recent review that specific training mainly starts after a rehabilitation period of 16 to 24 weeks. However, several studies have described accelerated rehabilitation after meniscus repair in which specific training already starts 8 to 12 weeks after meniscus repair [27]. But, none of these recommendations are supported by basic science or prior outcome research.

In this study, early rehabilitation after meniscus replacement included also a wide range of recommendations. The lack of consent is in accordance with the current literature which is characterized by the weak and inconsistent study situation [10, 17, 33, 34]. There is a consent to restrict early rehabilitation after meniscus replacement in contrast to the concepts for partial meniscectomy and even meniscus repair. The currently used rehabilitation protocols recommend reduced weight bearing to begin 4 weeks after surgery. All protocols involve full load bearing 8 weeks after meniscal replacement. Nevertheless, these results are compatible with already published recommendations after meniscus replacement in which restriction of weight bearing have also been described up to 8 weeks after surgery [10, 17, 35–38]. Even a mean return to full weight bearing after 6 weeks was confirmed by the findings of Rosso et al., who reviewed 55 studies investigating meniscus allograft transplantation [34], and by Filardo et al., who reviewed meniscus replacement by scaffolds [17]. Limited ROM was recommended in all patients with a minimum of 6 weeks after surgery and free ROM starting 9 weeks after surgery. These variations in early rehabilitation concepts are also reflected in the current literature; in that, full ROM is recommended from the 6^th^ postsurgical week onwards [10, 17, 34–36]. With regard to the use of braces, there is a consensus in the evaluated rehabilitation protocols on the use of an orthosis for additional external joint stabilization for 8 postoperative weeks, even if the current literature is lacking such evidence. A few published concepts recommend brace therapy for a period from 4 to 9 postoperative weeks [17, 35, 39], but Rosso et al. ascertained that there is no consensus in the postoperative use of an orthosis [34].

Nevertheless, the present study has some limitations which should be considered in the interpretation of its results. Because of the study design, the data stemmed from an investigation of currently used standardized rehabilitation protocols lack factors such as clinical outcome, patient compliance, or follow-up data. Also, technical details, such as patterns of meniscus lesions and details about the techniques of meniscus repair used and replacement, are missing. Thus, no further qualitative interpretation about patient outcome or best practice is possible. Additionally, the evaluated protocols cover the early period of rehabilitation up to 12 weeks, so regeneration and return to work/sport that require a longer period of rehabilitation are not covered by this data sample. In this way, this study just provides sufficient and novel information about the currently used rehabilitation protocols for the early postsurgical phase after meniscus surgery. These results do not give any evidence on the clinical results of the used protocols but provide a guideline for physiotherapists and clinicians about the mean values for the use of an orthosis, weight bearing, range of motion, and start of training after surgery and also show the variability of different recommendations in specific treatments.

## 5. Conclusion

The present study describes the variance of recommendations for the daily routine in early rehabilitation after partial meniscectomy, meniscus repair, and meniscus replacement recommended in German, Austrian, and Swiss orthopedic institutions. Overall, a wide range of recommendations exists for weight bearing, ROM, brace therapy, and mobilization, particularly after meniscus repair and meniscus replacement. However, the range seems to be in accordance with concepts and the data situation in the current literature, including a very early functional start of rehabilitation after partial meniscectomy and a rather restricted start after meniscus repair or replacement. Further high-quality research is required to investigate the evidence of the evaluated rehabilitation concepts and to create a general guideline on the different injury patterns and treatment options.

## Data Availability

The data used to support the findings of this study are available from the corresponding author upon reasonable request.
